# Development of Sucrose-Utilizing *Escherichia coli* Nissle 1917 for Efficient Heparosan Biosynthesis

**DOI:** 10.3390/metabo15060410

**Published:** 2025-06-18

**Authors:** Yaozong Chen, Zihua Wan, Zheng-Jun Li

**Affiliations:** State Key Laboratory of Green Biomanufacturing, National Energy R&D Center for Biorefinery, and Beijing Key Laboratory of Green Chemicals Biomanufacturing, Beijing University of Chemical Technology, Beijing 100029, China; 2022201227@buct.edu.cn (Y.C.); 2022201220@buct.edu.cn (Z.W.)

**Keywords:** heparosan, sucrose, carbon co-utilization, Nissle 1917

## Abstract

**Background/Objectives**: Heparosan is a component of the capsular polysaccharide in *Escherichia coli* K5 and *Pasteurella multocida* Type D. It shares a similar glycan structure with heparin and can be enzymatically modified to produce bioactive heparin. **Methods**: In this study, the probiotic strain *E. coli* Nissle 1917 (EcN), which naturally produces heparosan, was genetically engineered to utilize sucrose as a carbon source for growth while achieving high-yield heparosan biosynthesis. **Results**: By expressing the sucrose hydrolase genes *sacA* (from *Bacillus subtilis*) or *spI* (from *Bifidobacterium adolescentis*), EcN was enabled to utilize sucrose, achieving heparosan titers of 131 mg/L and 179 mg/L, respectively. Further metabolic engineering was performed to block the glycolytic and pentose phosphate pathways, thereby redirecting sucrose-derived glucose-6-phosphate and fructose-6-phosphate toward heparosan biosynthesis, while glycerol was supplemented as an auxiliary carbon source to support cell growth. Finally, the key biosynthesis genes *galU*, *kfiD*, and *glmM* were overexpressed, resulting in an engineered strain with a heparosan titer of 622 mg/L. **Conclusions**: This study represents the first successful engineering of EcN to utilize sucrose as the carbon source for growth, while achieving enhanced heparosan production through synergistic carbon source utilization. These findings establish a foundational strategy for employing this strain in the sucrose-based biosynthesis of other glycosaminoglycans.

## 1. Introduction

*Escherichia coli* Nissle 1917 (EcN) is a probiotic strain widely recognized for its safety and therapeutic efficacy in treating gastrointestinal disorders [1]. It can adhere to intestinal epithelial cells and continuously secrete bacteriocins that antagonize *Salmonella*, thereby inhibiting pathogen colonization and toxin production while maintaining gut microbiota homeostasis [2]. With its robust metabolic versatility and genetic tractability, EcN has emerged as a versatile engineered live biotherapeutic platform, enabling targeted delivery of anti-inflammatory molecules, antimicrobial agents, and metabolic modulators [3]. For example, engineered EcN can activate the immune system to induce potent and sustained tumor-specific antigen immune responses, serving as an antitumour vaccination platform [4]. Additionally, EcN was engineered to synthesize 3-hydroxybutyrate to alleviate intestinal inflammation [5], and to express epidermal growth factor to mitigate intestinal ulcers and promote epithelial repair [6].

Similar to *E. coli K5* and *Pasteurella multocida* Type D, EcN possesses the biosynthetic and secretory pathways for heparosan accumulation, a crucial glycosaminoglycan with the basic structure [-4)-β-D-glucuronic acid-(1→4)-α-D-N-acetylglucosamine-(1→]n [7,8,9]. Through chemoenzymatic modifications involving epimerization and sulfation, heparosan can be converted into the bioactive heparin. Heparin is a highly sulfated glycosaminoglycan widely used in clinical anticoagulation therapy. Currently, heparin production relies on extraction from animal tissues, which carries potential safety risks and supply chain vulnerabilities [10]. Therefore, developing animal-free heparin production methods is of significant importance. Among all currently known strains capable of naturally synthesizing heparosan, EcN represents the sole Generally Recognized as Safe (GRAS) microorganism. Metabolic engineering of this probiotic strain for enhanced heparosan production would lay the groundwork for environmentally sustainable heparin preparation [11].

Sucrose, a disaccharide composed of glucose and fructose, is particularly abundant in sugarcane and beet plants. As the predominant sweetener in the food industry and the major component of molasses, sucrose serves as an economical bulk carbon source for microbial fermentation due to its economic and environmental advantages [12]. Among wild-type *E. coli* strains, only a limited number possess native sucrose utilization capability [13]. Heterologous expression of the sucrose utilization operon *cscABC* coupled with glucaric acid biosynthetic genes in *E. coli* BL21 (DE3) yielded a recombinant strain producing 1.42 g/L glucaric acid from 10 g/L sucrose [14]. Expression of the sucrose hydrolase *sacA* in *E. coli* ZY0217 enabled 46.34 g/L lysine production using pretreated beet molasses as feedstock in fed-batch fermentation [15].

**Figure 1:**
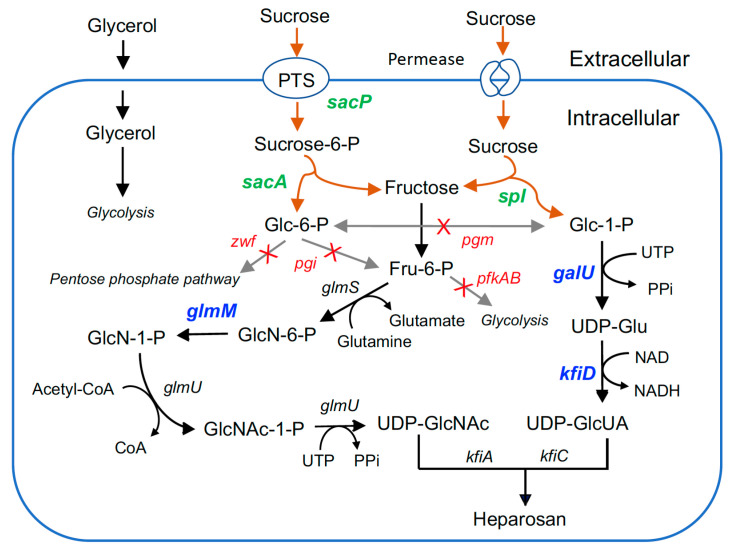
Metabolic engineering strategy for heparosan biosynthesis using sucrose and glycerol.

In the present work, the biosynthetic pathways for sucrose utilization and heparosan formation were rationally engineered in EcN (Figure 1). Initially, two distinct sucrose utilization pathways were expressed in EcN, and the effects of the sucrose transporter on cell growth and carbon utilization were investigated. More importantly, the endogenous pathways of glucose-6-phosphate and fructose-6-phosphate into cell biomass were blocked, ensuring that the majority of glucose and fructose from sucrose were used for heparosan production while glycerol was employed to support cell growth. To further increase the product titer and yield, the key enzymes in the heparosan biosynthesis pathways were also engineered.

## 2. Materials and Methods

### 2.1. Bacterial Strains and Plasmid Construction

The bacterial strains and plasmids utilized in this work are detailed in Table 1. Plasmid assembly and propagation were performed using *E. coli* DH5α, whereas genome editing for heparosan production was implemented in the probiotic strain *E. coli* Nissle 1917 (EcN). Bacterial cultures were routinely grown in Luria-Bertani (LB) medium (10 g/L tryptone, 5 g/L yeast extract, 10 g/L NaCl) under aerobic conditions at 37 °C. To ensure plasmid retention, antibiotics were added to the medium at the following concentrations: Ampicillin (100 µg/mL), Kanamycin (50 µg/mL), and Chloramphenicol (34 µg/mL).

The sucrose phosphorylase gene *spI* from *Bifidobacterium adolescentis* (NCBI accession WP_195394230.1) was synthesized by BGI Co., Ltd., Beijing, China. The sucrose-6-phosphate hydrolase gene *sacA* and sucrose transporter component gene *sacP* were cloned from *Bacillus subtilis* 168, while the glucose-1-phosphate uridylyltransferase gene *galU*, UDP-glucose dehydrogenase gene *kfiD*, glucosamine-fructose-6-phosphate aminotransferase gene *glmS*, phosphoglucosamine mutase gene *glmM*, and bifunctional UDP-N-acetylglucosamine pyrophosphorylase/glucosamine-1-phosphate N-acetyltransferase gene *glmU* were amplified from EcN. Target gene fragments were PCR-amplified using primer pairs listed in Supplementary Table S1. Gene assembly was performed through Gibson isothermal recombination to achieve scarless integration of DNA fragments. For individual gene expression, amplified sequences were directionally inserted into pEC-XK99E. Simultaneous expression of multiple genes was accomplished by combinatorial insertion into the plasmid backbone.

### 2.2. Chromosomal Gene Knockout in EcN

Targeted gene disruptions (Δzwf, ΔpfkB, ΔpfkA, Δpgi, and Δpgm) were achieved through λ-Red recombinase-assisted genomic recombination, as previously described [16]. Antibiotic resistance markers flanked by 50-bp homology arms were PCR-amplified from pKD13 template DNA using gene-specific primer sets (Supplementary Table S2). The resulting linear knockout cassettes were introduced via electroporation into EcN strains harboring the arabinose-inducible pKD46 plasmid encoding λ-Red recombinase.

### 2.3. Shake Flask Cultivation for Heparonsan Production

Initial seed cultures were cultivated in LB medium at 37 °C with agitation at 200 rpm for a duration of 14 h. For assessing sucrose utilization, 2% (*v*/*v*) of the seed culture was inoculated into 250 mL baffled Erlenmeyer flasks, each containing 50 mL of M9 minimal medium supplemented with 5 g/L sucrose. Heparosan biosynthesis was conducted in shake flask experiments using R medium, which was composed of the following (per liter): 4 g of Bacto tryptone, 6 g of yeast extract, 13.5 g of KH_2_PO_4_, 4 g of (NH_4_)_2_HPO_4_, 1.4 g of MgSO_4_·7H_2_O, 1.7 g of citrate, 0.1 g of thiamine hydrochloride, and 10 mL of a prepared trace elements solution. The trace elements solution was made by dissolving the following compounds in one liter of deionized water: 2 g of CaCl_2_, 2.2 g of ZnSO_4_·7H_2_O, 0.5 g of MnSO_4_·4H_2_O, 10 g of FeSO_4_·7H_2_O, 0.02 g of Na_2_B_4_O_7_·10H_2_O, 1 g of CuSO_4_·5H_2_O, and 0.1 g of (NH_4_)_6_Mo_7_O_2__4_·4H_2_O. A mixed carbon source comprising sucrose and glycerol was utilized. The medium contained sucrose at an initial concentration of 2 g/L and glycerol at 20 g/L.

### 2.4. Analytical Methods

During shake flask cultivation, samples were taken at regular intervals to evaluate both cellular growth and metabolite profiles. Cell density was monitored by recording absorbance at 600 nm (OD_600_). Collected culture samples were subjected to centrifugation at 8000× *g* for 10 min to pellet the cells, after which the supernatants were passed through 0.22 μm syringe filters. These clarified samples were then analyzed via high-performance liquid chromatography (HPLC) to assess carbon substrate consumption. The HPLC system used (LC-20A, Shimadzu, Japan) included an Aminex HPX-87H ion-exchange column (Bio-Rad, Hercules, CA, USA) coupled with a refractive index detector. Chromatographic separation was carried out at 55 °C using 5 mM sulfuric acid as the mobile phase, delivered at a constant rate of 0.6 mL/min.

The procedure for isolating and quantifying heparosan followed previously established protocols [9]. In short, 50 mL of culture broth was centrifuged at 10,000× *g* for 10 min, and the resulting supernatant was mixed with four times its volume of pre-chilled ethanol, followed by incubation at −20 °C overnight to allow for polysaccharide precipitation. The precipitate was collected by centrifugation (10,000× *g*, 10 min, 4 °C), air-dried, and reconstituted in deionized water. Any remaining particulates were removed via additional centrifugation. Heparosan content was determined using the carbazole assay, with D-glucuronic acid serving as the calibration standard [9]. In brief, 1 mL of sample was reacted with 5 mL of H_2_SO_4_ at 100 °C for 10 min. After cooling on ice, 250 μL of 0.125% (*w*/*v*) carbazole reagent was added and incubated in boiling water for 15 min. The chromogenic product was quantified at 530 nm using a spectrophotometer to calculate heparosan concentration.

## 3. Results

### 3.1. Establishment of Sucrose Utilization Pathways in EcN

Microorganisms primarily employ two sucrose assimilation systems. First, sucrose is transported into cells via the phosphotransferase system (PTS) while being simultaneously phosphorylated to generate sucrose-6-phosphate, followed by hydrolase-catalyzed cleavage into fructose and glucose-6-phosphate [17]. Second, sucrose can be internalized through the permease system and subsequently cleaved either by sucrase into fructose and glucose or by sucrose phosphorylase into fructose and glucose-1-phosphate, with all resulting products ultimately entering the glycolytic pathway [18].

**Figure 2:**
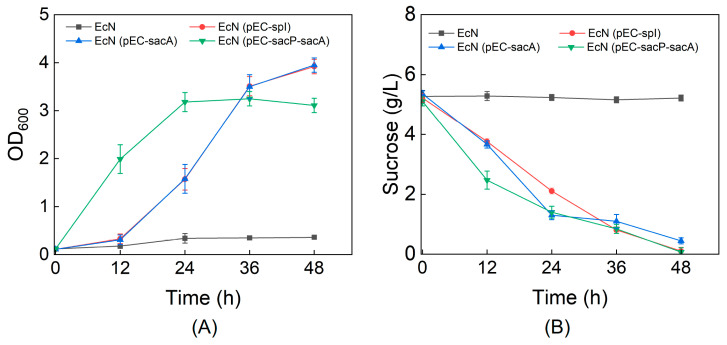
Cell growth (**A**) and sucrose utilization (**B**) profiles of engineered EcN strains cultivated in M9 medium.

The wild-type EcN strain cannot utilize sucrose as a sole carbon source for growth. To enable sucrose assimilation, we engineered two distinct pathways in EcN: (1) expressing sucrose-specific PTS enzyme II component SacP and sucrose-6-phosphate hydrolase SacA from *B. subtilis*; and (2) expressing sucrose phosphorylase SpI from *Bifidobacterium adolescentis* (Figure 1). After introducing the respective plasmids into EcN, we evaluated the recombinant strains’ growth and substrate consumption in M9 minimal medium containing 5 g/L sucrose as the sole carbon source. As shown in Figure 2, while the wild-type strain completely lacked sucrose utilization capability, recombinant EcN strains expressing either *spI* or *sacA* alone could grow on sucrose and nearly depleted 5 g/L sucrose within 48 h. This suggests that EcN may possess nonspecific transporters on its cell membrane that facilitate sucrose uptake, which remains to be elucidated in future studies. Compared to the strain expressing *sacA* alone, the recombinant strain co-expressing *sacA* with the PTS component *sacP* exhibited enhanced cell growth and sucrose utilization efficiency.

### 3.2. Heparosan Production Using Sucrose as the Carbon Source

**Figure 3:**
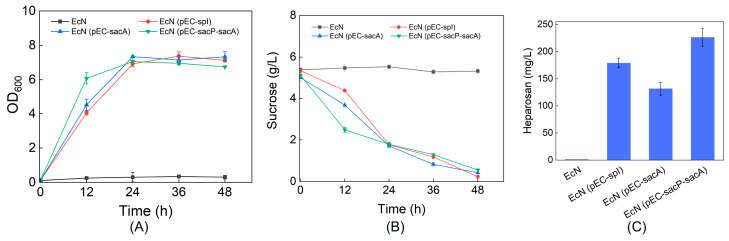
Cell growth (**A**), sucrose utilization (**B**), and heparosan production (**C**) profiles of engineered EcN strains cultivated in R medium.

We next evaluated heparosan production in recombinant strains harboring the sucrose utilization pathways using R medium supplemented with sucrose as the carbon source. Compared with M9 minimal medium, R medium, containing 6 g/L yeast extract and 4 g/L Bacto tryptone, provided organic nitrogen sources that significantly enhanced bacterial growth rate and maximal OD_600_, yet sucrose consumption remained unchanged. The recombinant strains EcN (pEC-spI), EcN (pEC-sacA), and EcN (pEC-sacP-sacA) achieved heparosan titers of 179 mg/L, 131 mg/L, and 226 mg/L, respectively (Figure 3). These results demonstrate that both engineered sucrose utilization pathways can effectively support heparosan biosynthesis, with production levels slightly higher than those obtained when the wild-type EcN strain utilizes glucose as the carbon source [9]. In addition, the expression of the PTS component SacP could internalize extracellular sucrose in its phosphorylated form. Compared to strains expressing only SacA, the additional expression of SacP produces more heparosan.

### 3.3. Improved Heparosan Production Using Mixed Carbon Sources

To further enhance heparosan production, we engineered the central carbon metabolic pathways through targeted genetic modifications: (1) deletion of the glucose-6-phosphate dehydrogenase gene *zwf* to prevent glucose-6-phosphate diversion into the pentose phosphate pathway; (2) knockout of phosphofructokinase genes *pfkA* and *pfkB* to block fructose-6-phosphate entry into glycolysis; (3) disruption of the phosphoglucose isomerase gene *pgi* to eliminate the interconversion between glucose-6-phosphate and fructose-6-phosphate (Figure 1). These modifications ensured that the two monosaccharides derived from sucrose catabolism were exclusively channeled into their respective UDP-sugar biosynthetic pathways for subsequent polymerization into heparosan. Glycerol was supplemented as an auxiliary carbon source to support bacterial growth.

**Figure 4:**
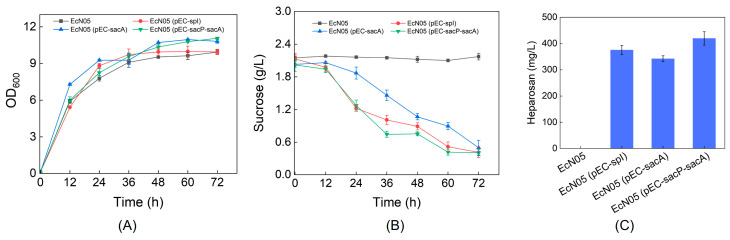
Improved heparosan production through carbon flux redirection. (**A**) Cell growth; (**B**) Sucrose consumption; (**C**) Heparosan production.

Following this metabolic engineering strategy, we constructed recombinant strain EcN05 and introduced the sucrose-metabolizing plasmids. When cultured in R medium with 2 g/L sucrose and 20 g/L glycerol as the carbon sources, the heparosan production titers of EcN05 (pEC-spI), EcN05 (pEC-sacA), and EcN05 (pEC-sacP-sacA) reached 375 mg/L, 342 mg/L, and 419 mg/L, respectively, representing near a two-fold increase compared to the control strain without glycolytic pathway engineering (Figure 4). Notably, the disruption of glycolytic pathways significantly reduced sucrose consumption to approximately 1.5 g/L while dramatically improving the heparosan yield. Specifically, the EcN05 (pEC-spI) strain achieved a yield of 0.28 g heparosan/g sucrose, marking an approximately 7-fold enhancement over the unmodified parental strain.

### 3.4. Deletion of Phosphoglucomutase to Improve Heparosan Production

**Figure 5:**
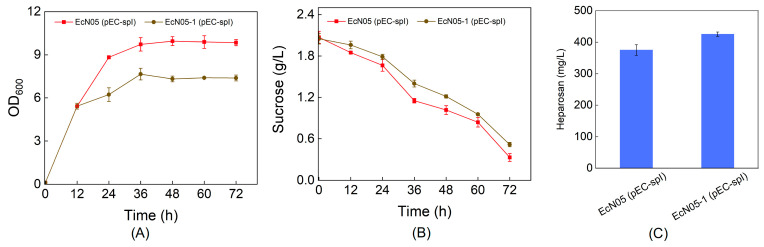
Effect of *pgm* deletion on heparosan production. (**A**) Cell growth; (**B**) Sucrose consumption; (**C**) Heparosan production.

We subsequently focused on the sucrose phosphorylase pathway, which enables efficient sucrose utilization in EcN through a single gene expression. In this pathway, sucrose is cleaved into fructose and glucose-1-phosphate. Since the endogenous phosphoglucomutase catalyzes the interconversion between glucose-1-phosphate and glucose-6-phosphate [19], we hypothesized that deleting the *pgm* gene might reduce the backflow of glucose-1-phosphate to glucose-6-phosphate, thereby directing more carbon flux toward heparosan biosynthesis (Figure 1). This experimental design evaluates the maximum theoretical advantage achievable by inhibiting glucose-1-phosphate isomerization. As shown in Figure 5, disruption of the phosphoglucomutase moderately impaired the growth of strain EcN05-1. This observation suggests that, despite glycerol serving as an auxiliary carbon source for biomass formation, the cells still require a certain amount of glucose-6-phosphate for biosynthesis of cellular components. Regarding heparosan production, the titer increased by approximately 50 mg/L in the *pgm*-deficient strain.

### 3.5. Overexpression of Key Enzymes to Improve Heparosan Production

**Figure 6:**
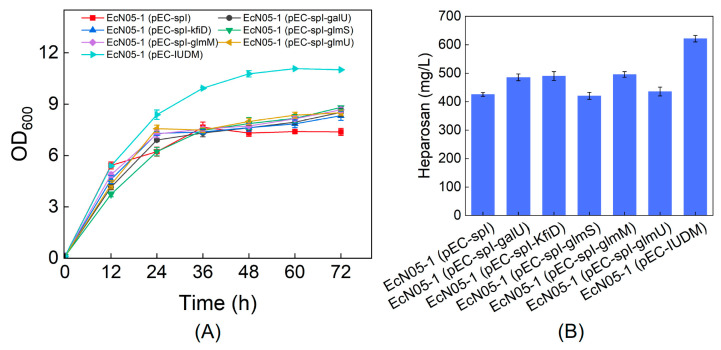
Effect of key enzyme overexpression on heparosan production. (**A**) Cell growth; (**B**) Heparosan production.

To further enhance heparosan production, we finally evaluated the impact of overexpressing critical biosynthetic enzymes in EcN05-1. Targeted genes comprised *galU* (glucose-1-phosphate uridylyltransferase), *kfiD* (UDP-glucose dehydrogenase), *glmS* (glutamine-fructose-6-phosphate aminotransferase), *glmM* (phosphoglucosamine mutase), and *glmU* (bifunctional UDP-N-acetylglucosamine pyrophosphorylase/glucosamine-1-phosphate N-acetyltransferase). The individual expression of *galU*, *kfiD*, and *glmM* significantly enhanced heparosan biosynthesis, with the production levels increasing by approximately 60 mg/L. In contrast, expression of *glmS* or *glmU* failed to demonstrate substantial improvement in heparosan titer (Figure 6). These findings suggest that the UDP-glucuronic acid biosynthesis branch probably serves as the rate-limiting step in heparosan production.

Subsequently, the three positively influential genes *galU*, *kfiD*, and *glmM* were co-overexpressed to evaluate the effects on cell growth and heparosan production. The results showed that EcN05-1 harboring plasmid pEC-IUMD exhibited significantly enhanced biomass accumulation. UDP-N-acetylglucosamine functions as an essential precursor for peptidoglycan synthesis, while UDP-GlcA participates in exopolysaccharide production [20,21]. Thus, the growth promotion may be attributed to the dual metabolic roles of these nucleotide sugars. Remarkably, the combinatorial expression of *galU*, *kfiD*, and *glmM* also substantially increased heparosan production to 622 mg/L, representing an approximately 50% improvement compared to the control strain EcN05-1 (pEC-spI) (Figure 6).

## 4. Discussion

Sucrose, as a disaccharide, requires decomposition into monosaccharides prior to its assimilation through the glycolytic pathway. This process demonstrates more gradual kinetics compared to monosaccharide utilization, which may help to mitigate carbon overflow metabolism caused by excessive carbon sources [22]. In this study, we successfully established two sucrose utilization routes in EcN, a one-step pathway using SpI and a two-step PTS-based route using SacP and SacA. Although EcN lacks native sucrose metabolism genes, our results suggest that it possesses nonspecific transporters, enabling limited sucrose uptake, as recombinant strains expressing only sucrose hydrolase or phosphorylase enzymes could still consume sucrose.

In the heparosan biosynthetic pathway, UDP-glucuronic acid is derived from glucose-6-phosphate, while UDP-N-acetylglucosamine originates from fructose-6-phosphate (Figure 1). Notably, these two critical precursors, glucose-6-phosphate and fructose-6-phosphate, can be simultaneously generated through sucrose degradation in equimolar quantities. This stoichiometric balance is particularly advantageous since heparosan’s polysaccharide backbone requires alternating polymerization of UDP-glucuronic acid and UDP-N-acetylglucosamine. Therefore, using sucrose as the carbon source inherently maintains metabolic flux balance between the two parallel biosynthetic branches for heparosan production.

Redirecting carbon flux by knocking out *zwf*, *pfkAB*, and *pgi* further decoupled biomass growth from heparosan biosynthesis, minimizing competition for intermediates. Glycerol supplementation served as an effective energy and biomass carbon source, maintaining cell viability while sucrose-derived intermediates were channeled toward heparosan. The ~7-fold increase in yield achieved in the engineered strain EcN05 (pEC-spI) validates the efficacy of this decoupling strategy (Figure 4). Further enhancement was achieved by deleting *pgm* to prevent the back-conversion of glucose-1-phosphate to glucose-6-phosphate. While growth was modestly impaired, likely due to decreased availability of glucose-6-phosphate for essential biosynthesis, the heparosan titer improved, confirming more carbon was redirected to product formation (Figure 5). Finally, overexpression of key biosynthetic enzymes demonstrated that GalU, KfiD, and GlmM are rate-limiting steps. Their simultaneous overexpression not only improved heparosan titer but also enhanced biomass formation (Figure 6).

The synthesis and export of heparosan are governed by the gene products of the *kps* locus. In *E. coli* K5, this locus is regulated by a complex network of transcriptional controls [23]. In EcN, glucose has been shown to repress heparosan production, while the cyclic AMP (cAMP)–cAMP receptor protein (CRP) complex binds to the upstream region of the region 3 promoter, thereby enhancing the transcription of the heparosan biosynthetic gene cluster [24]. Further transcriptomic analysis of the *kps* locus under mixed carbon source conditions may help elucidate the transcriptional regulatory mechanisms and enhance the expression strength of genes involved in heparosan biosynthesis, thereby contributing to further improvements in production titer. Moreover, traditional heparosan biosynthesis typically uses glucose as the primary carbon source. Sucrose is abundant in waste molasses, and glycerol can be obtained as a by-product of biodiesel production [25,26]. Employing sucrose and glycerol as substitute carbon sources for heparosan production may thus offer economic advantages over glucose.

In earlier research, multiple metabolic engineering approaches have been applied to boost heparosan production in EcN. By fine-tuning the expression of critical pathway genes, including *galU*, *kfiD*, *glmM*, *kfiA*, and *kfiC*, titers of up to 1.29 g/L were achieved in shake-flask experiments. Further enhancement was accomplished under fed-batch conditions, reaching titers as high as 11.50 g/L [9]. Additionally, a strategy based on antibiotic-driven chromosomal evolution was used to amplify the *kps* gene cluster. This method enabled a 24-fold increase in *kps* copy number, resulting in a production titer of 9.1 g/L in fed-batch culture [8]. In this study, EcN was engineered to utilize sucrose, followed by rewiring the central carbon metabolism pathway. Therefore, carbon fluxes between biomass formation and heparosan synthesis were decoupled to boost heparosan biosynthesis. In-depth investigation of the rate-limiting steps in heparosan biosynthesis, coupled with precise modulation of key enzyme expression, could potentially further enhance heparosan production yields.

While the current study relies on high-copy-number plasmids for gene overexpression, this approach may present challenges for long-term or large-scale bioreactor cultivation. Plasmid instability under these conditions could result in progressive productivity loss and elevated downstream processing costs. To overcome these limitations, future work should focus on chromosomal integration of the target expression cassettes in EcN. For optimal performance, such integration strategies should target genomic high-expression loci or employ strong promoters to compensate for the reduced gene dosage in single-copy configurations [27]. In addition, future research would benefit from investigating fermentation parameters such as mixed carbon source feeding strategies in bioreactors to further improve the production level.

## 5. Conclusions

This study establishes an engineered *E. coli* Nissle 1917 platform for heparosan biosynthesis using sucrose as the carbon source. By reconstructing sucrose utilization pathways (*sacP/sacA* or *spI*) and strategically blocking competitive glycolytic routes (Δ*zwf*, Δ*pfkAB*, and Δ*pgi*), we achieved efficient channeling of sucrose carbon flux into UDP-sugar biosynthesis and enabled balanced precursor supply for heparosan polymerization. Combinatorial overexpression of rate-limiting enzymes *galU*, *kfiD*, and *glmM* synergistically enhanced UDP-sugar availability and heparosan titer, ultimately yielding 622 mg/L heparosan in shake flasks. The developed probiotic platform presents a sustainable and animal-free alternative for heparosan manufacturing, with potential adaptability for other glycosaminoglycans.

## Figures and Tables

**Table 1:** Strains and plasmids used in this study.

Strains/Plasmids	Description	Source
Strains		
*E. coli* DH5α	Wild-type strain for gene cloning	Takara
*E. coli* Nissle 1917	Wild-type strain for heparosan production	DSMZ *
*Bacillus subtilis* 168	Wild-type strain for cloning *sacA* and *sacP*	DSMZ
*E. coli* EcN05	Nissle 1917 Δzwf ΔpfkB ΔpfkA Δpgi	This study
*E. coli* EcN05-1	Nissle 1917 Δzwf ΔpfkB ΔpfkA Δpgi Δpgm	This study
Plasmids		
pKD13	Plasmid harboring Kan^R^ and FLP recognition target	Yale CGSC **
pKD46	λ-Red recombinase expression helper plasmid	Yale CGSC
pCP20	FLP recombinase helper plasmid	Yale CGSC
pEC-XK99E	Expression vector, *trc* promoter, Kan^R^	Sangon
pEC-spI	pEC-XK99E derived, harboring *spI*	This study
pEC-sacA	pEC-XK99E derived, harboring *sacA*	This study
pEC-sacP-sacA	pEC-XK99E derived, harboring *sacP* and *sacA*	This study
pEC-spI-galU	pEC-XK99E derived, harboring *spI* and *galU*	This study
pEC-spI-kfiD	pEC-XK99E derived, harboring *spI* and *kfiD*	This study
pEC-spI-glmS	pEC-XK99E derived, harboring *spI* and *glmS*	This study
pEC-spI-glmM	pEC-XK99E derived, harboring *spI* and *glmM*	This study
pEC-spI-glmU	pEC-XK99E derived, harboring *spI* and *glmU*	This study
pEC-IUDM	pEC-XK99E derived, harboring *spI*, *galU*, *kfiD*, and *glmM*	This study

* DSMZ, German Collection of Microorganisms and Cell Cultures. ** Yale CGSC, The *E. coli* Genetic Stock Center.

## Data Availability

The original contributions presented in this study are included in the article/Supplementary Materials. Further inquiries can be directed to the corresponding author.

## Supplementary Materials

The following supporting information can be downloaded at: https://www.mdpi.com/article/10.3390/metabo15060410/s1, Table S1: Oligonucleotides used for plasmid construction in this study; Table S2: Oligonucleotides used for gene deletion in this study.
