# Supplementary material for: Development of Sucrose-Utilizing Escherichia coli Nissle 1917 for Efficient Heparosan Biosynthesis
Source: Metabolites. 2025 Jun 18;15(6):410. doi: 10.3390/metabo15060410 (PMC12194944; doi:10.3390/metabo15060410)
Supplement: Supplementary file 1 [file metabolites-15-00410-s001.zip › metabolites-3660413-supplementary.pdf]

Supplementary materials

Table S1 Oligonucleotides used for plasmid construction in this study.

Primers	Sequence (5’-3’)	Notes
pEC-spI-galU-GF	tactagagaaagaggagaaatactagatggctgccattaatacgaaagtcaaaaaag	Construction of pEC-spI-galU
pEC-spI-galU-GR	gcaggtcgacttacttcttaatgcccatctcttcttcaagc	
pEC-spI-galU-ZF	taagaagtaagtcgacctgcaggcatgc	
pEC-spI-galU-ZR	ctagtatttctcctctttctctagtatctagattatgcaacaaccggcg	
pEC-spI-kfiD-GF	ctagatactagagaaagaggagaaatactagatgttcgg	Construction of pEC-spI-kfiD
pEC- spI-kfiD-GR	aggtcgacttagtcacatttaaacaaatcgcgactatagactttg	
pEC- spI-kfiD-ZF	aaatgtgactaagtcgacctgcaggcatgc	
pEC- spI-kfiD-ZR	cctctttctctagtatctagattatgcaacaaccggcg	
pEC-spI-glmS-GF	gcataatctagaatgtgtggaattgttggcgc	Construction of pEC-spI-glmS
pEC-spI-glmS-GR	gcaggtcgacttactcaaccgttaccgattttgcc	
pEC-spI-glmS-ZF	ggttgagtaagtcgacctgcaggcatgc	
pEC-spI-glmS-ZR	ccacacattctagattatgcaacaaccggcg	
pEC-spI-glmM-GF	ctagatactagagaaagaggagaaatactagatgagtaatcg	Construction of pEC-spI-glmM
pEC-spI-glmM-GR	gcaggtcgacttaaacggcttttactgcatcggc	
pEC-spI-glmM-ZF	agccgtttaagtcgacctgcaggcatgc	
pEC-spI-glmM-ZR	cctctttctctagtatctagattatgcaacaaccggcg	
pEC-spI-glmU-GF	ctagagtcgacatgttgaataatgctatgagcgtagtgatc	Construction of pEC-spI-glmU
pEC-spI-glmU-GR	gcatgcctgcagttactttttctttatcggacgacgcc	
pEC-spI-glmU-ZF	aaaagtaactgcaggcatgcaagcttg	
pEC-spI-glmU-ZR	attcaacatgtcgactctagattatgcaacaaccg	
pEC-IU-KfiD-GF	gaagtaagtcgacttgacaattaatcatccggctcgtataatg	Construction of pEC-IUDM
pEC-IU-KfiD-GR	atgcctgcagttagtcacatttaaacaaatcgcgactatagactttg	
pEC-IU-KfiD-ZF	atgtgactaactgcaggcatgcaagcttg	
pEC-IU-KfiD-ZR	ttgtcaagtcgacttacttcttaatgcccatctc	
pEC-IU-KfiD-glmM-GF	ctaactgcagtactagagaaagaggagaaatactagatgagtaatcg	
pEC-IU-KfiD-glmM-GR	gcttgcatgcttaaacggcttttactgcatcggc	
pEC-IU-KfiD-glmM-ZF	agccgtttaagcatgcaagcttggctgttttg	
pEC-IU-KfiD-	ttctctagtactgcagttagtcacatttaaacaaatcgc	

glmM-ZR		
pEC-VF	ttgcgccgacatcataacgg	Verification of pEC-derived plasmids
pEC-VR	atcagaccgcttctgcgttc	

Table S2 Oligonucleotides used for gene deletion in this study.

Primers	Sequence (5’-3’)
zwf-F	atggcggtaacgcaaacagcccaggcctgtgacctggtcattttcggcgcgaaaggcgacattcc ggggatccgtcgacc
zwf-R	tactcaaactcattccaggaacgaccgtcacgggtaatcatcgccaccgaggcaacggggtgtag gctggagctgcttcg
zwf-VF	gcgcgcttttaccgtaatcg
zwf-VR	gtaaccggagctcatagggc
pfkB-F	atggtacgtatctatacgttgacacttgcgccctctcttgatagcgcaacaattaccccgattccggg gatccgtcgacc
pfkB-R	tagcgggaaaggtacgcgtaaattttttgcgtatcgtcatgggagcacaggcgtgttccgtgtaggc tggagctgcttcg
pfkB-VF	cactttccgctgattcggtg
pfkB-VR	acaggttggtggtgattccc
pfkA-F	atgattaagaaaatcggtgtgttgacaagcggcggtgatgcgccaggcatgaacgccgcaattcc ggggatccgtcgacc
pfkA-R	taatacagtttttttcgcgcagtccagccagtcgcctttgaacggacgcttcatgttttcgtgtaggctg gagctgcttcg
pfkA-VF	agagccagacccgcattttg
pfkA-VR	aagcgcatcaggcctttttg
pgi-F	atgaaaaacatcaatccaacgcagaccgctgcctggcaggcactacagaaacacttcgatattcc ggggatccgtcgacc
pgi-R	ttaaccgcgccacgctttatagcggttaatcagaccattggtcgagctatcgtggctgcgtgtaggc tggagctgcttcg
pgi-VF	tgcgctagcgcaggtagtac
pgi-VR	gcgcggggaattagtgatgg
pgm-F	atgaaactgcaggggggtaattttcgatctggatggagtaatcacagataccgcgcatctgattccgg ggatccgtcgacc
pgm-R	ctatacgttttgccagaaggccgataaccgcggccaggtcagggagtccgttgagggcaatgtag gctggagctgcttcg
pgm-VF	atgacggtatccacgctgcc
pgm-VR	tgcatcttcaggccaaacgc
